# Quick Surgical Diagnoses

**Published:** 1898-02

**Authors:** 


### Quick Surgical Diagnoses.

A man of genteel breeding and intellectual force remarked recently in Boston, that he wears sewed to his undershirt a card with this inscription: "My appendix has been cut out." And he gave this reason for his action: "You see these are the palmy knifing days of the surgeon. If a man falls in a fit or faints, or is disguised mentally by a drug, and is carried consequently to a hospital, the surgeon operates on him for appendicitis without delay."—*Practical Druggist.*

